# virusMED: an atlas of hotspots of viral proteins

**DOI:** 10.1107/S2052252521009076

**Published:** 2021-09-28

**Authors:** HuiHui Zhang, Pei Chen, Haojie Ma, Magdalena Woińska, Dejian Liu, David R. Cooper, Guo Peng, Yousong Peng, Lei Deng, Wladek Minor, Heping Zheng

**Affiliations:** a Hunan University College of Biology, Bioinformatics Center, Hunan 410082, People’s Republic of China; b Biological and Chemical Research Centre, Chemistry Department, University of Warsaw, Żwirki i Wigury 101, 02-089 Warsaw, Poland; c University of Virginia, Charlottesville, VA 22908, USA; d Hunan Provincial Key Laboratory of Medical Virology, People’s Republic of China

**Keywords:** virus hotspots, viral protein structures, epitopes, antiviral drugs, DrugBank, viral metal proteins, virusMED database

## Abstract

Metal binding sites, antigen epitopes and drug binding sites are the hotspots in viral proteins that control how viruses interact with their hosts. virusMED (*virus* *M*etal binding sites, *E*pitopes and *D*rug binding sites) is a rich internet application based on a database of atomic interactions around hotspots in 7041 experimentally determined viral protein structures. 25306 hotspots from 805 virus strains from 75 virus families were characterized, including influenza, HIV-1 and SARS-CoV-2 viruses. Just as Google Maps organizes and annotates points of interest, virusMED presents the positions of individual hotspots on each viral protein and creates an atlas upon which newly characterized functional sites can be placed as they are being discovered. virusMED contains an extensive set of annotation tags about the virus species and strains, viral hosts, viral proteins, metal ions, specific antibodies and FDA-approved drugs, which permits rapid screening of hotspots on viral proteins tailored to a particular research problem. The virusMED portal (https://virusmed.biocloud.top) can serve as a window to a valuable resource for many areas of virus research and play a critical role in the rational design of new preventative and therapeutic agents targeting viral infections.

## Abstract 2

virusMED (Virus Metal binding sites, Epitopes and Drug binding sites) is a rich internet application based on a database of atomic interactions around hotspots in 7041 experimentally determined viral protein structures.

## 1. Background   

Viruses are ubiquitous in our world (Paez-Espino *et al.*, 2019 ▸), and human infections by viruses can potentially result in serious diseases (Tang *et al.*, 2020 ▸). Viral components are insufficient to support the complete virus-replication cycle, and therefore viruses can only grow and replicate in living host cells. Viral infections are initiated when virus surface proteins interact with receptors on the host cell, followed by invasion of the target cell through a different mechanism. Primary thera­peutic antiviral drugs (Sosa *et al.*, 2018 ▸) that can interact with the receptor-binding sites or the crucial domains of other viral components (Siramshetty *et al.*, 2018 ▸) include small-molecule compounds (Hermann, 2016 ▸), peptides (Vilas Boas *et al.*, 2019 ▸) and antibodies that serve as potential therapeutic agents (Klasse & Moore, 2020 ▸). In rare cases, there are also broad-spectrum antivirals that are known to target homologous viral proteins across species (Verbruggen *et al.*, 2018 ▸).

The primary prophylactic agents are vaccines that can imitate viruses and stimulate an immune response to provide protection from viral infections (Vetter *et al.*, 2018 ▸). The currently used types of vaccines mainly include chemically inactivated viruses, attenuated viruses (Sizikova *et al.*, 2019 ▸), recombinant protein antigens (Mascola & Fauci, 2020 ▸), replication-defective viruses, virus-like particles (Fuenmayor *et al.*, 2017 ▸) and nucleic acid-vectored vaccines (Lee, Arun Kumar *et al.*, 2018 ▸; Sandbrink & Shattock, 2020 ▸). However, traditional vaccines are often futile against viruses that mutate frequently, such as HIV and influenza (Oscherwitz, 2016 ▸). The imperfect design of vaccine immunogens, the inherent flexibility of viral antigens, and pathogen mutations can result in antigenic changes that reduce the protection efficacy of the vaccine-induced specific immune responses. Rational, structure-guided vaccine antigen design is a promising strategy to increase the breadth and potency of vaccines (Chen *et al.*, 2019 ▸).

The hotspots on viral protein structures described herein are local sequence and structural features that facilitate the binding of small molecules or other macromolecules. The atomic interactions between a viral protein and its binding partners include hydrophobic interactions, hydrogen bonds, salt bridges and coordination bonds. Its repertoire of genome products highly orchestrate the viral pathogenesis process. Identifying and characterizing the hotspots on the viral proteins and the corresponding receptor-binding interfaces is a vital step in further research into the molecular mechanisms of the viral pathogenesis process and the rational design of molecular agents for use in diagnostic and therapeutic procedures against viruses. Information on hotspots on the structures of viral proteins can be used in the drug-design process to identify lead compounds and drive subsequent optimization. Hotspot analysis can also aid in the design of vaccine immunogens with optimal antigenicity (Lee *et al.*, 2015 ▸). The complete genomic information and macromolecular structural information on virtually all structural and nonstructural components in the SARS-CoV-2 genome is highly informative for facilitating the development of preventative and therapeutic agents (Grabowski *et al.*, 2021 ▸). For example, NSP3 or 3c-like proteinase structures may guide the development of drugs for the treatment of SARS, while spike glycoprotein-based immunogens are designed to stimulate specific immune responses against SARS-CoV-2 (Hsieh *et al.*, 2020 ▸).

Metal binding sites are hotspots on viral proteins that serve either as an architectural site or a catalytic site (Chaturvedi & Shrivastava, 2005 ▸), yet their importance is often overlooked. Metal-containing compounds offer a viable alternative for targeting unusual structural or chemical features on viral proteins that are otherwise inaccessible to organic compounds (de Paiva *et al.*, 2020 ▸). Many metallodrugs can inhibit HIV by targeting different proteins, such as magnesium or zinc targeting integrase (Carcelli *et al.*, 2014 ▸), cobalt targeting nucleocapsid protein (Louie & Meade, 1998 ▸) and gold targeting reverse transcriptase and protease enzymes (Fonteh & Meyer, 2009 ▸). Recent studies have shown that ranitidine bismuth citrate (a metallodrug) can inhibit both the ATPase and helicase in SARS-CoV-2 (Yuan *et al.*, 2020 ▸). The correct modeling of metal binding sites in macromolecular structures requires knowledge from multiple disciplines, including biology, coordination chemistry and crystallography (Zheng, Cooper *et al.*, 2017 ▸; Yao & Moseley, 2019 ▸). There are plenty of databases archiving the metal binding sites in macromolecular structures (Lin *et al.*, 2016 ▸; Ireland & Martin, 2019 ▸), which all rely on the structural information contained in the Protein Data Bank (PDB; Burley *et al.*, 2017 ▸). However, none of these resources focuses specifically on the presence and relevance of metal binding architectures in viral proteins.

Epitopes are the hotspots where antibodies bind to viral proteins. Vertebrates possess an adaptive immune system that can recognize a wide range of exotic immunogens from various pathogens. The corresponding ‘antigenic cluster’ of immunogens on the viral proteins can trigger immune responses. Therefore, precise and in-depth knowledge of ‘antigenic clusters’ is critical for the development of diagnostics and therapeutics targeting infectious, allergic and autoimmune diseases and carcinoma (Dhanda *et al.*, 2019 ▸). More specifically, the information on epitopes allows the rational design of fine-tuned or truncated immunogens (Deng *et al.*, 2018 ▸), epitope-focused vaccines (Correia *et al.*, 2014 ▸), or optmization of antibody cocktail therapy (Starr *et al.*, 2021 ▸). For example, identifying the epitopes of a 2019-nCoV vaccine helped to elucidate its working mechanism and led to its production for clinical use (Lucchese, 2020 ▸). Moreover, the recent successful development of a monoclonal antibody cocktail therapy against SARS-CoV-2 by Regeneron Pharmaceuticals Inc. is based mainly on a critical analysis of the epitopes recognized by various neutralizing antibody candidates (Hansen *et al.*, 2020 ▸). Sotrovimab, an emerging effective treatment for COVID-19, used structural biology to guide improvements to make an antibody that can neutralize all known SARS-CoV-2 strains and other SARS viruses (Starr *et al.*, 2021 ▸).

Information about experimentally determined epitopes, their specific antibodies and the corresponding immune reactions are archived in the Immune Epitope Database (IEDB; Peters *et al.*, 2005 ▸), which includes linear epitopes and structural characteristics of nonlinear conformational epitopes if available (Vita *et al.*, 2019 ▸). The sister web service IEDB-AR provides tools that can predict and analyze hypothetical epitopes (Dhanda *et al.*, 2019 ▸). The data and associated tools from the IEDB have facilitated the rational design of epitope-based therapies and immuno-focused vaccination strategies. For example, algorithms have been developed to decrease the undesired immunogenicity of vaccines (Dhanda *et al.*, 2018 ▸; Fleri *et al.*, 2017 ▸). The conformational epitope database (CED) is another resource that systematically archives known conformational epitopes (Huang & Honda, 2006 ▸). An in-depth investigation of epitopes on a viral protein in the context of other types of hotspots at a species level would further advance our knowledge regarding the rational design of epitope-based therapies and vaccines.

Small-molecule binding sites are hotspots that can serve as the target of antiviral drugs (Wishart *et al.*, 2006 ▸, 2018 ▸) and antiviral peptides (Jhong *et al.*, 2019 ▸). For example, the antiviral compound benztropine interacts with the envelope glycoprotein of the Ebola virus to inhibit viral infection (Ren *et al.*, 2018 ▸). While we define drugs as small-molecule compounds collected by the DrugBank database, other small-molecule binders observed in complex with viral protein structures are also cataloged here because they may represent potential binding sites. The information on ‘ligand-binding’ viral proteins can then be instrumental for the design of antivirals. Small-molecule agents are often used to inhibit the activities of a viral enzyme or block the interaction between viral proteins and their binding partners. Drug binding sites on viral proteins often correlate with metal binding sites and epitopes on the same protein (Varadi *et al.*, 2020 ▸; Panda *et al.*, 2020 ▸). A database of binding pockets harboring small-molecule compounds specifically on viral proteins would provide a framework for the design of candidate drugs targeting viral infections, yet no current resource is readily available for this purpose.

Many very good databases have been developed that focus on drug–gene interactions (Cotto *et al.*, 2018 ▸) and the known resistance to antimicrobial drugs (Doster *et al.*, 2020 ▸). Some information about epitopes, small-molecule binding sites and metal binding sites of viral proteins can be found scattered among half a dozen other resources. Databases have been developed with a special focus on virulence factor–host interactions (Sayers *et al.*, 2019 ▸) or virus–host interactions (Zhang *et al.*, 2021 ▸). There are also databases investigating intramolecular interactions for a specific class of virus, such as the database of flu–drug interactions (Zhang *et al.*, 2017 ▸) and the database of prion–drug interactions (Lee, Lee *et al.*, 2018 ▸). Databases of coronavirus protein structures provide invaluable structural information for developing therapeutic agents against SARS-CoV-2 (Gowthaman *et al.*, 2021 ▸). However, no currently available database systematically indexes the hotspots and intermolecular interactions on the structures of all viral proteins, which could provide a framework for the design of candidate drugs targeting viral infections.

Here, we describe the development of the virusMED database, which encompasses metal binding site, epitope and drug binding site information about all viral proteins that have a determined structure in a single resource. Our database provides one-stop, combinatory representations and annotations of hotspots on the structural proteomes of viruses. It presents logically essential information for the rational design of preventative, diagnostic and therapeutic procedures against viral infections.

## 2. Materials and methods   

### 2.1. Virus taxonomy data   

The set of viral protein structures was downloaded using the 22 April 2021 version of the PDB (Burley *et al.*, 2017 ▸), with the corresponding metadata stored in a local implementation of the PDBj Mine RDB version 2 (Kinjo *et al.*, 2017 ▸). The source of each macromolecule chain is obtained from the NCBI Taxonomy ID in the metadata of the mmCIF file header, defined as either the source of the gene or the source organism in cases of naturally sourced samples. The classification information of all viruses is obtained from the 1 January 2021 version of the NCBI taxonomy database (Federhen, 2012 ▸). In cases where obsolete taxonomy has been used in the PDB files, the obsolete taxonomy IDs are mapped to the corresponding updated taxonomy IDs using the merged.dmp table downloaded from the same version of the NCBI taxonomy database. The possible virus taxonomy IDs are defined as those tagged with division IDs corresponding to viruses, synthetic constructs and environmental samples. Chimeric polypeptide strands associated with multiple taxonomy IDs are divided into multiple fragments according to each taxonomy ID. If a commonly used expression tag is determined for any of the taxonomy IDs, the taxonomy ID of the other fragment is used as the taxonomy ID of the chimeric polypeptide strand.

The information on each virus species is obtained from the International Committee on Taxonomy of Viruses (ICTV) database (Lefkowitz *et al.*, 2018 ▸), together with the virus family, the kingdom/phylum of the general cellular host and the Baltimore classification [dsDNA, ssDNA, dsRNA, ssRNA(+), ssRNA(−), ssRNA, ssRNA/dsDNA-RT] of each virus species. We map each species in the ICTV to the taxonomy IDs in the NCBI taxonomy database using the corresponding RefSeq IDs reported in each ICTV entry. For the few entries that lack RefSeq IDs, the ICTV species name is used to match the species, genus and family from the NCBI taxonomy database until a match is found, and the corresponding NCBI Taxonomy ID is linked to that ICTV entry. 12 ICTV species from the ICTV belong to the *Semotivirus* genus in the *Belpaoviridae* family. *Belpaoviridae* is a new family confirmed by ICTV in 2018, containing the genus *Semovirus* (previously included in the *Metaviridae* family). The NCBI taxonomy database has not implemented a corresponding mechanism to catalog this type of virus (Krupovic *et al.*, 2018 ▸), and the NCBI Taxonomy IDs are left blank in the database.

**Figure 1:**
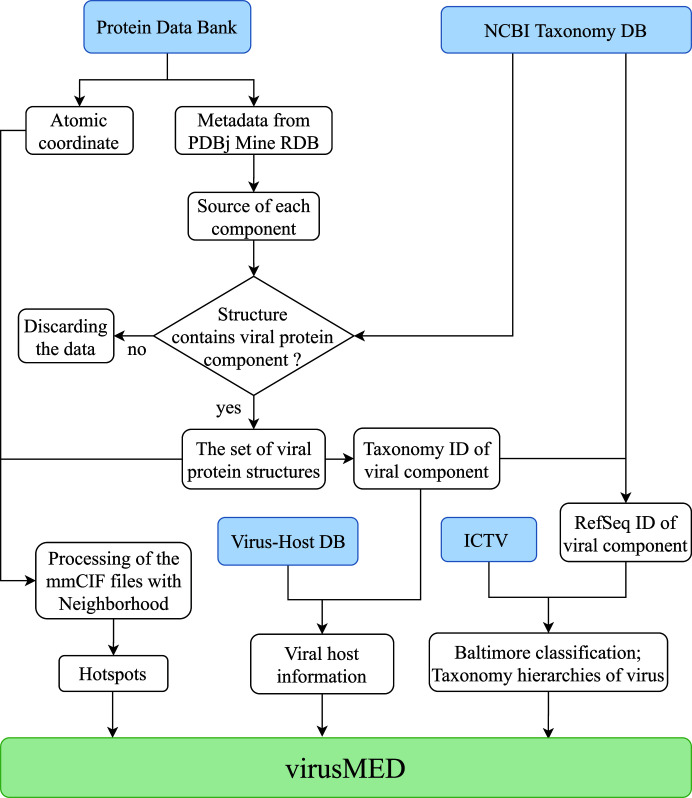
Flowchart illustrating the data-acquisition and integration process in virusMED. Data sources are highlighted in blue.

Each virus species reported in the ICTV may represent multiple virus strains. The classification hierarchies and the scientific name of each virus strain are obtained from the NCBI taxonomy database using the corresponding taxonomy IDs. The known species of cellular hosts susceptible to the virus strain of interest are obtained from the Virus–Host DB (Mihara *et al.*, 2016 ▸). Quite often, an NCBI Taxonomy ID from the metadata of the mmCIF file header represents a specific virus strain that may not precisely match an ICTV species or an entry from the Virus–Host DB. Each virus strain is mapped to the corresponding virus species from either the ICTV or the Virus–Host DB using the NCBI taxonomy hierarchy information. In most cases a match to an ICTV species or an entry from the Virus–Host DB can be found at the species level, while in rare cases a match may also be found at the genus or family level. We have provided a flowchart to illustrate the mapping from a PDB structure to a Virus–Host DB entry using NCBI Taxonomy ID and to an ICTV entry using both the NCBI Taxonomy ID and the RefSeq sequence ID (Fig. 1 ▸).

### 2.2. Processing of structural data   

Preprocessing of the PDB files of viral protein structures includes scanning and summarizing the residue types and the completeness of the structural models in the coordinates section in the mmCIF files. We use a version of the Neighborhood database (Zheng *et al.*, 2008 ▸) to process all PDB structures that contain at least one viral protein component. This is a relational database that stores the interactions between the atoms and residues of all modeled viral protein structures, together with other PDB-derived information such as the name and source of each biological component and the viral protein enzyme classification number (EC number). The Neighborhood database provides an effective way to store, query and classify the intermolecular interactions for a diverse set of hotspots in viral protein structures, including the metal binding sites, epitope and drug binding sites.

### 2.3. Metal binding sites   

The intermolecular interactions between metal ions and viral proteins are stored as the coordinating bonds and represent the constructed metal binding sites. Free metal ions with no significant coordinating bonds are removed from the data set. The polypeptide chain that forms the most coordinating bonds with the subject metal ion is characterized and used as the viral source of the metal binding site. The type of metal ion and the enzymatic classification number (EC number) of each polypeptide chain coordinating the metal ion are used to annotate each metal binding site in the database. The quality of each metal binding site is evaluated using the previously described algorithm used in the validation of magnesium binding sites in nucleic acid structures (Zheng *et al.*, 2015 ▸). Since the previous algorithm was only tested for magnesium ions, the validation parameters are adapted to be applicable to all metal binding sites.

### 2.4. Epitopes   

Complex structures containing both viral components and nonviral components are distinguished using the NCBI Taxonomy ID from the metadata of the mmCIF file header as described in Section 2.1. Nonviral components in a complex structure can be the binding partner of the viral protein from its cellular host or an immunoglobulin or nanobody. PDB chains with nonviral components in the viral protein structure complex possessing greater than 30% sequence similarity to a known B-cell antibody, nanobody or T-cell receptor sequence are indexed in a data set of antibodies. The set of antibodies is used to select the viral proteins or glycoproteins within the Neighborhood database to identify all antibody–antigen interfaces. Epitopes interacting with a B-cell antibody or nanobody are annotated as B-cell epitopes, while epitopes interacting with T-cell receptors are annotated as T-cell epitopes. Epitopes defined by the antibody–antigen interface are also annotated using the source organisms used to produce antibodies.

Many viral antigen chains possess covalently linked glycosyl­ation features assigned as individual glycan chains. These covalently linked glycan chains are considered to be an integral part of the viral protein as long as a characteristic covalent bond is identified for N- or O-glycosylation. Interfaces between the antibodies and the viral proteins or glycoproteins include mostly hydrophobic interactions and polar inter­actions such as hydrogen bonds and salt bridges. We characterize the conformational epitope of the viral protein as the collection of viral amino acids and glycan residues involved in the intermolecular interactions. The paratope is the collection of antibody amino acids and glycan residues located in the antibody–antigen interface.

### 2.5. Drug binding sites   

The intermolecular interactions between small molecules and viral proteins are stored as the hydrophobic interactions, hydrogen bonds and other polar interactions between the small-molecule compound and the corresponding binding pocket. The polypeptide chain with the most extensive interactions with the small molecule is considered to be the viral source of the drug binding site. The chemical structure of each small molecule in the database is compared with that of the drug molecule reported in the DrugBank (Wishart *et al.*, 2006 ▸, 2018 ▸) using the open-source cheminformatics software *RDKit* (Lovrić *et al.*, 2019 ▸). The approval status of the drug is annotated using information from the DrugBank as ‘FDA approved’, ‘in DrugBank’ or ‘not in DrugBank’ for each small molecule. The corresponding generic drug name, drug groups and drug ATC codes are also annotated for each compound in the DrugBank. Small molecules are annotated as known cofactors, porphyrin-ring-like compounds, metal-organic compounds and other organic compounds.

### 2.6. Database and web-server implementation   

All data are processed and organized into a PostgreSQL Database Management System. The backend of the virusMED database uses PostgreSQL 10.15 (database server). The web server is deployed using an Ubuntu Linux virtual machine running Nginx 1.14.0 and Gunicorn 20.0.4. The interface components of the website are designed and implemented using the Django template engine 3.1.4. Molecular graphics on the view page use HTML5 as implemented in the NGL Javascript library. Static molecular graphics on the home page are produced using *PyMOL* (Schrödinger), although visual art with viruses was purchased from https://www.veer.com/. virusMED has been tested in several popular web browsers, including Google Chrome 89.0.4389.82, Mozilla Firefox 87.0, Apple Safari 13.0.2 and Microsoft Edge 89.0.774.75. The styles of the web interface are optimized using the Bootstrap 4.5.0 library to accommodate both large computer screens and small screens on handheld devices. The virusMED webserver is accessible via https://virusmed.biocloud.top/. The underlying data, such as the list of virus strains, viral proteins and hotspots, are available for print or bulk download as flat files in the format of the user’s choice. The relevant data can be downloaded in PDF, Excel or CSV format, which allows the convenient import as a relational database table into *Excel* or other widely used database-management systems.

## 3. Data statistics, web interface and utilities   

### 3.1. Content of the virusMED database   

The current version of the virusMED database comprises 25 306 hotspots from 7041 viral protein structures. The structures are from 805 virus strains and account for 75 distinctive virus families, covering more than 40% of the known virus families reported in the ICTV. Throughout the text, we use the term (candidate) ‘drug binding site’ to describe the small-molecule binding pocket on the viral protein structure that a drug can potentially target. Drug binding sites are the most frequently encountered hotspots on a viral protein structure, currently contributing 10 977 hotspots and 2470 unique hotspots from 4951 viral protein structures. Metal binding sites are found in 67 virus families, compared with 60 virus families for drug binding sites. Conformational epitopes are the least commonly observed hotspots in the virusMED database. There are 5593 epitopes and 1186 unique hotspots in 1174 viral protein structures, encompassing the viral protein surfaces that have so far been mapped by antibody–antigen complex structures from 209 virus strains from 31 virus families (Table 1 ▸). Redundant protein structures from the same virus strain are not included in the calculation of unique proteins. Redundant hotspots from the same viral protein are not included in the calculation of unique hotspots. Even if a structure is considered redundant based on sequence similarity, the hotspots within the structure may be unique due to the sample preparation resulting in the formation of different structural complexes.

**Figure 2:**
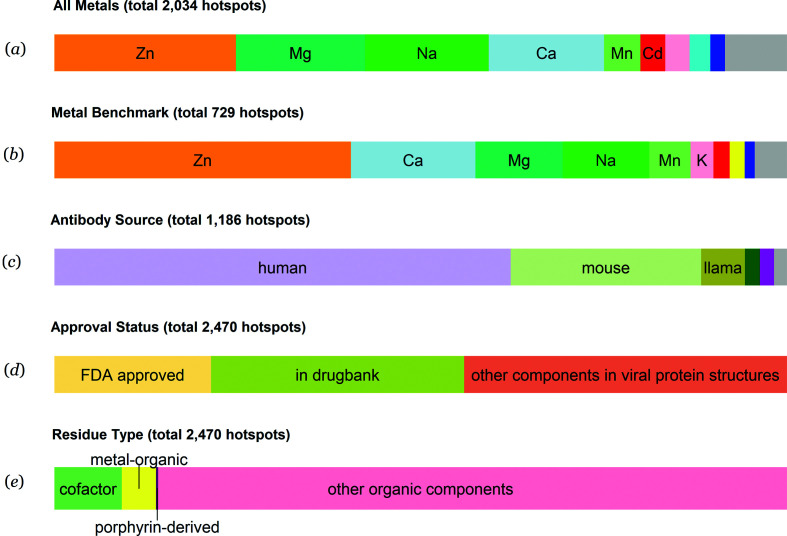
Statistics of nonredundant hotspots in the virusMED database. (*a*) Distribution of metal binding sites in viral protein structures; pink, cyan, blue and gray represent K, Hg, Ni and others, respectively. (*b*) Distribution of metal binding sites in the benchmark data set, which were defined as metal binding sites that passed the validation procedures described in Section 2; red, yellow, blue and gray represent Cd, Fe, Ni and others, respectively. (*c*) Distribution of source organisms that generate antibodies; green, purple and gray represent rabbit, monkey and others, respectively. (*d*) Distribution of drug approval status. (*e*) Distribution of types of small-molecule compounds in viral protein structures. Only nonredundant depositions are presented. Redundancy in a PDB deposition does not necessarily imply redundancy in the hotspots.

Zinc binding sites are the most common metal binding sites presented in the virusMED database and represent more than 25% of all metal binding sites. Magnesium, sodium, calcium and manganese binding sites comprise another 55% of the metal binding sites [Figs. 2 ▸(*a*) and 2 ▸(*b*)]. Antibodies from human sources constitute more than 60% of all antibodies in virus–antibody complex structures, immediately followed by antibodies produced by mice and nanobodies from llamas [Fig. 2 ▸(*c*)]. More than 50% of all compounds in the database are entries in the DrugBank, with more than one-third having been approved by the FDA [Fig. 2 ▸(*d*)]. Most of the ligands observed in drug binding sites are from the general category ‘organic compounds’. Known cofactors and metal–organic compounds encompass only a small fraction of all drug binding sites [Fig. 2 ▸(*e*)].

**Figure 3:**
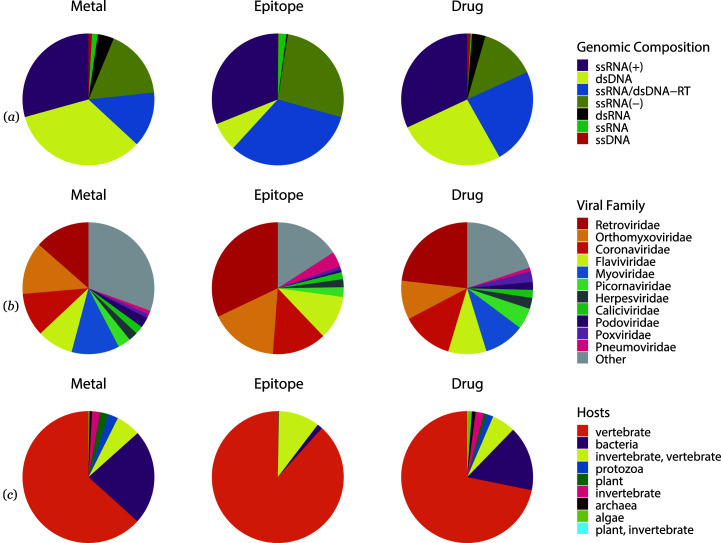
Statistics of the Baltimore classifications of the genomic composition of the genetic materials, virus family and viral hosts for different types of hotspots in the virusMED database. (*a*) Distribution of the Baltimore classification of viruses that contain metal binding sites, epitopes and drug binding sites. (*b*) Distribution of the top 11 virus families that contain metal binding sites, epitopes and drug binding sites. (*c*) Distribution of viral hosts that contain metal binding sites, epitopes and drug binding sites.

The ssRNA(+) viruses contribute the largest number of hotspots, followed by dsDNA, ssRNA/dsDNA-RT and ssRNA(−). The same trend applies for the number of metal binding sites and drug binding sites, yet dsDNA viruses contribute fewer epitopes when compared with ssRNA/dsDNA-RT or ssRNA(−) viruses [Fig. 3 ▸(*a*)]. Further investigation of the top 11 most common virus families in hotspot-containing viral protein structures reveals that the *Retroviridae* family was the most thoroughly studied virus family for all three types of hotspots, followed by *Corona­viridae*, *Orthomyxoviridae* and *Flaviviridae* [Fig. 3 ▸(*b*)]. The *Myoviridae* family is the third most studied virus family in the case of metal binding sites and drug binding sites. Viruses that infect vertebrates contribute the most hotspots, followed by viruses that infect bacteria for metal and drug binding sites or by viruses that infect invertebrates for epitopes [Fig. 3 ▸(*c*)]. There is a noteworthy number of cases where a virus is present in multiple hosts. The distribution of viral hosts represented in virusMED reflects the availability of structural and immunological information. Within the PDB, viruses that infect vertebrates, bacteria and invertebrates/vertebrates are amply represented, but viruses that infect plants and other hosts are less represented. Also, the availability of structural information for viruses that infect different types of hosts is dependent on the type of virus hotspot. Epitope information is also absent for many viral proteins. For example, viruses with conformational epitopes are distributed in only four kingdoms/phyla of the general cellular host [Fig. 3 ▸(*c*)].

Not all viruses are equally represented from the structural perspective. A survey of the diversity of viral proteins in the virusMED database indicates that bacteriophage T4 possesses a genome that encodes more than 300 gene products and is the best-studied virus (Miller *et al.*, 2003 ▸). It is also a commonly used tool in many biotechnology applications (Hess & Jewell, 2020 ▸; Table 2 ▸). Despite the relatively recent emergence of SARS-CoV-2 and its much smaller genome compared with T4 phage, structural biology studies of SARS-CoV-2 proteins are marching forward at an unprecedented pace (Grabowski *et al.*, 2021 ▸) and hotspots from 16 distinct proteins have been characterized (Brzezinski *et al.*, 2021 ▸). HIV-1 is another virus that has attracted long-standing interest and exhibits 23 protein clusters as defined by the 30% sequence-identity cutoff (Abram *et al.*, 2010 ▸). Other virus strains that have more than ten protein clusters structurally determined include Vaccinia virus, Acanthamoeba polyphaga mimivirus and Influenza A virus (Table 2 ▸).

### 3.2. Browsing hotspots in virusMED   

The virusMED home page contains several entry points that allow public access to the data in the database. The user can either browse by hotspots or browse by viruses. The ‘Browse by Hotspots’ options feature three entry points corresponding to metal binding sites, epitopes and drug binding sites. The ‘Browse by Viruses’ set of options features three other entry points and allows researchers to browse by virus strains, viral genome composition (as defined by the Baltimore classification) and the kingdom/phylum of the viral host.

#### 3.2.1. Browse by Hotspots   

The ‘Metal’ option links to an elemental periodic table that allows the user to browse all the entries in the database that contain a specific type of metal ion. Additionally, eight other tags are pre-defined to enable easy access to a particular subset of metals in the viral protein structures: common metals (Na, Mg, K, Ca, Mn, Fe, Co, Ni, Cu, Zn), alkali metals, alkaline earth metals, all transition metals, transition metals in the 4th/5th/6th period and post-transition metals. The epitope option uses an option list to select all epitopes, B-cell epitopes, T-cell epitopes or epitopes recognized by an antibody produced from specific antibody-yielding organisms of interest: rabbit, cow, llama, monkey, mouse, chimpanzee, human, camel, rat and synthetic. The drug option uses an option list to select either drugs with a certain approval status in the DrugBank or a type of small molecule (common cofactor, porphyrin-ring derivatives, metal–organic compound or other organic compounds). Shortcuts are also provided at the top right of the navigation bar to allow quick access to all metal binding sites, epitopes or drug binding sites.

#### 3.2.2. Browse by Viruses   

The ‘Virus strains’ option links to a complete list of virus strains annotated by the number of protein clusters that have been determined to date in the structural virome, similar to that in Table 2 ▸. Polypeptide chains with greater than 30% sequence similarity are considered to belong to the same protein cluster. Both the ‘Genome Composition’ and ‘Viral Hosts’ options link to a subset of virus strains from the complete list of virus strains. Genome Composition provides labels according to the Baltimore classification [dsDNA, ssDNA, dsRNA, ssRNA(+), ssRNA(−), ssRNA and ssRNA/dsDNA-RT], while ‘Viral Hosts’ provides labels according to a list of cellular hosts at the kingdom/phylum level (protozoa, fungi, plant, vertebrate, invertebrate, algae, bacteria and archaea). Selecting an entry from the list of virus strains leads to a summary page for that strain. Shortcuts are also provided for a representative set of the 21 most frequently encountered virus families, including 15 that infect humans, other vertebrates or invertebrates, three that infect bacteria, two that infect plants and one that infects protozoa.

#### 3.2.3. Summary of a virus strain and a viral protein   

**Figure 4:**
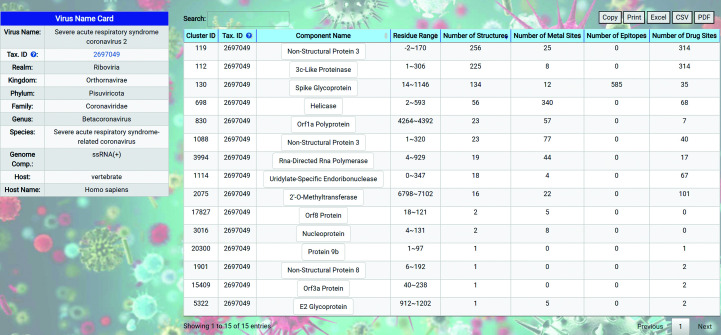
The virus-strain summary page for SARS-CoV-2.

The summary page for a virus strain lists the taxonomy and other summary information about the virus strain in the left panel as the ‘Virus Name Card’ and shows a list of distinct protein clusters in the right panel. The number of structures, number of metal binding sites, number of epitopes and number of drug binding sites are summarized and shown for each protein cluster. The summary page for SARS-CoV-2 is shown for illustration purposes (Fig. 4 ▸). The list of distinct protein clusters shown in the right panel of the summary page for a virus strain summarizes the protein types with hotspots for a single virus strain. Annotations of the sequence length, start position and ending position of each protein structure are added to differentiate between viral proteins that have the same component name but represent distinct domains or fragments of the same viral protein. Selecting an entry from the list of individual viral proteins leads to a summary page for that viral protein consisting of the summary information and a representative structure for the viral protein in the left panel and three lists of hotspots of that viral protein in the right panel. Each of the three lists in the right panel corresponds to the metal binding sites, epitopes and drug binding sites that have been observed in experimental viral protein structures from the PDB. The pages are dynamically scaled, so the left and right panels may be stacked on smaller screens.

### 3.3. Searching hotspots in the virusMED   

The virusMED interface provides several filters in the left control panel and a list of hotspots in the right data panel. The control panel also provides interactive filters, which allow the user to select a subset of a given list of hotspots. General filters applied to all three types of hotspots include the genome composition, taxonomy of viruses (family) and viral hosts. Custom filters are also implemented for a specific type of hotspot: the type of metals filter is implemented for metal binding sites, the type of epitopes and the species of antibody-producing organism are implemented for epitopes, and the class of compounds and drug approval status filters are implemented for drug binding sites. A filter limits the results to a nonredundant subset of hotspots by default, but the option to view all hotspots is also available. The number of hits for each filter combination is updated in real time upon selection. The table can be sorted on the results of each filter, either by alphabetic name or by the number of occurrences. Holding down the Ctrl button on the keyboard allows the user to select multiple entries simultaneously within the same filter. A user-defined combination of annotation tags provides an efficient and versatile way to screen subsets of hotspots on viral proteins tailored to the specific research needs.

The data panel contains the list of hotspots with the PDB code, chain ID and the corresponding residues of interest. The genome composition, family, host and component name of the viral protein are also shown for each hotspot. For metal binding sites, the metal ion name and validation parameters are shown, and the ‘bench’ tag indicates whether the metal binding site passed the validation criteria. For epitopes, the component names and source are shown for both the viral protein and the antibody. The list of residues constituting the epitopes and paratopes is shown in an abbreviated list. For drug binding sites, the name, type of compound and drug approval status for the small molecule are shown. An input text box allows the user to search the entries in the list of hotspots using a user-specified keyword. The list of hotspots will update itself in real time upon typing the keywords. Once a hotspot of interest is located, the user can choose to view detailed information on the hotspot as described in the next section.

### 3.4. Viewing hotspots in the virusMED   

**Figure 5:**
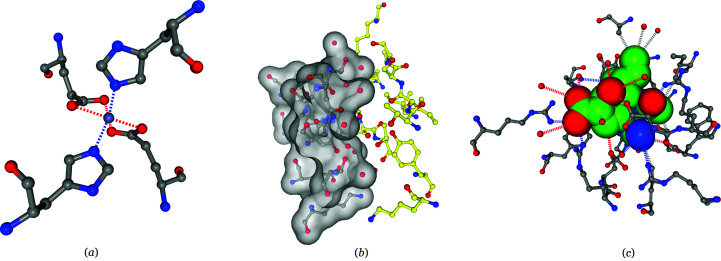
The influenza A virus as an example to show the appearance of the interactive molecular viewer for the three hotspot categories. (*a*) An example of a metal binding site: PDB entry 6lks, chain *K*, Zn, 3.24 Å. (*b*) An example of the epitope on hemagglutinin: PDB entry 4xnq, antibody (chain *B*)–antigen (chain *C*, surface), 2.0 Å. (*c*) An example of a drug binding site on neuraminidase: PDB entry 4mx0, chain *A*, peramivir, 2.1 Å.

The hotspot-viewing window includes both metadata and detailed information about the hotspot. The metadata includes information about the virus strain, the viral protein component and the experimental structure containing the hotspot. The detailed information about the hotspot includes a 3D NGL molecule-viewer window and a table that contains the list of individual intermolecular interactions involved in the hotspot. Several checkboxes are implemented to allow the users to toggle various annotation elements (such as the labels, surface *etc.*). Additional checkboxes are also provided to show only a subset of interactions (hydrophobic interactions, polar interactions and other interactions). Examples of the metal binding sites, epitopes and drug binding sites are shown to illustrate the interactive molecular viewer (Fig. 5 ▸).

## 4. Discussion and future work   

### 4.1. virusMED presents different types of hotspots in a unified format   

To facilitate structural–functional relationship research in viruses, the virusMED platform presents a wide variety of information about and annotations of functionally critical sites in experimentally determined viral protein structures. This allows researchers to quickly and easily see information about hotspots (metal binding sites, antigen epitopes and drug binding sites) that affect virus–host interaction in the proper structural context that has previously not been available within one resource.

Although some of the information in virusMED is also contained in individual databases, the siloed nature of these resources makes it difficult to gather all the relevant information. We performed a survey to compare existing databases with virusMED and have summarized their corresponding features in Supplementary Table S1. Databases that collate detailed information about metals and their coordination patterns include MetalPDB (Andreini *et al.*, 2013 ▸) and databases for specific metals, such as the zinc binding site database Zincbind (Ireland & Martin, 2019 ▸). Databases centered on epitopes include the T-cell epitope database EPIMHC (Reche *et al.*, 2005 ▸), the B-cell epitope database Epitome (Schlessinger *et al.*, 2006 ▸), the conformational epitope database CED (Huang & Honda, 2006 ▸) and the multifaceted database IEDB (Peters *et al.*, 2005 ▸). IEDB is the most frequently visited database and primarily focuses on humans, nonhuman primates and other animal species. Databases that describe the interactions and affinities between drug-like molecules and drug-target proteins include BindingDB (Chen *et al.*, 2001 ▸) and PSMDB (Wallach & Lilien, 2009 ▸). VIPERdb (Montiel-Garcia *et al.*, 2021 ▸) is a database that stores virus capsid structures and various virus-specific information. To date, there is no comprehensive molecular database of viral protein hotspots from all known structures of viruses, and virusMED provides a comprehensive and complementary resource that will add a powerful weapon to the virologists’ arsenal in the battle against viruses.

The virusMED database allows the simultaneous investigation of multiple hotspots and their structural relationship on the same viral protein or even on the same viral proteome, which serves as an atlas giving researchers an unprecedented advantage in the rational design of immunogens, antibodies or antiviral drugs. For example, inspecting the correlation between drug binding sites, epitopes and metal binding sites on the same protein would result in the rational design of combination medication cocktails targeting different hotspots or even different types of hotspots on viral proteins, while systematic mapping of epitope sites on the viral protein allows the rational design of truncated immunogens or epitope-focused vaccines.

### 4.2. Future work   

The virus classification information on the name of the strain in the database is obtained from the NCBI taxonomy database using the NCBI Taxonomy ID reported in the metadata of the mmCIF file header. These virus IDs are not always reported at the strain level; some are reported at the species level, hindering the direct acquisition of detailed information on the strain level of each virus. For viruses that are not annotated at the strain level, manual curation and routine updates will be needed to label them with reference to the published literature on the viral protein structure.

It has been shown that drug compounds may be effective across different virus species (Verbruggen *et al.*, 2018 ▸). For example, zanamivir, an FDA-approved drug used to treat flu, is also effective against Human respirovirus 3 from the *Paramyxoviridae* family (Table 3 ▸). More details of the individual strain and protein targeted by each drug are available in Supplementary Table S2. Currently, the server only allows hotspot comparison within the same strain as identified by the NCBI Taxonomy ID. Useful features in further development would include comparison of the hotspots across strains within the same species, such as hemagglutinin and neuraminidase from influenza virus, or even within the same virus family, such as the viral surface glycoproteins from different flavivirus strains. Comparing the same hotspot among homologous proteins could assist in other applications, such as vaccine-antigen design and active-site analysis.

virusMED can serve as a blueprint for an Advanced Information System (AIS; Zheng, Porebski *et al.*, 2017 ▸) pertaining to viruses, as it aims to provide novel analysis and gather information from more specialized databases and web resources. Collection of this information is essential to prepare for any future biomedical threats and challenges (Grabowski *et al.*, 2021 ▸) by providing comprehensive and instantaneous avenues of research. The internal flexibility of virusMED allows the easy addition of features to increase the breadth of available data. For example, external links may be expanded to include genomic annotations across species within the same families, orders or even classes (Grazziotin *et al.*, 2017 ▸). Utilities that allow systematic classification and comparative algorithms that can evaluate the local similarity in metal binding sites (Zheng *et al.*, 2015 ▸), epitopes and drug binding sites in lieu of global sequence identities would prove to be useful in a wide variety of applications. The availability of hotspot-comparison utilities will also allow us to implement a tool for the submission of a new protein structure or 3D motif to search the database for known binding sites for metals, drugs or antibodies.

The new functionalities listed above are not only our ideas but are also suggestions from early users of the virusMED database. The creation and publication of databases such as virusMED is only the first step in changing the way that researchers will interact with scientific information in the future. A database that is not updated and kept current is not only obsolete but can actually hinder scientific progress. Similarly, maintaining internal consistency and consistency with external databases and resources is a critical step towards transformation to an AIS. For this reason, we are planning to perform frequent updates to a test server and release a major update of the virusMED database to the stable production server semi-annually.

## 5. Abbreviations   

The abbreviations used are as follows. virusMED, virus Metal binding sites, Epitopes and Drug binding sites; FDA, Food and Drug Administration; CED, conformational epitope database; PDB, Protein Data Bank; PDBj, Protein Data Bank Japan; NCBI, National Center for Biotechnology Information; ICTV, International Committee on Taxonomy of Viruses; IEDB, Immune Epitope Database; IEDB-AR, Immune Epitope Database Analysis Resource; ATC code, Anatomical Thera­peutic Chemical code; EC number, enzyme classification number; HIV, Human immunodeficiency virus 1; SARS-CoV-2, Severe acute respiratory syndrome coronavirus 2; NSP3, nonstructural protein 3; ATPase, adenosine triphosphatase; AIS, Advanced Information System.

## Supplementary Material

Supplementary Tables. DOI: 10.1107/S2052252521009076/be5290sup1.pdf

## Figures and Tables

**Table 1:** Hotspots statistics of the virusMED database

Hotspot feature	No. of virus families	No. of virus species	No. of virus strains	No. of PDB structures	No. of hotspots	No. of unique proteins	No. of unique hotspots
Metal	67	271	525	2699	8736	821	2034
Epitope	31	80	209	1174	5593	329	1186
Drug	60	233	514	4951	10977	869	2470
All hotspots	75	346	805	7041	25306	1520	5690
Data from ICTV	169	6587	—	—	—	—	—

**Table 2:** The list of the most thoroughly studied viruses from the structural point of view Diversity of hotspot-containing viral protein structures in the virusMED database. Virus strains with more than ten distinct hotspot-containing protein structures are shown.

ICTV virus species name	NCBI Taxonomy ID	NCBI taxonomy strain scientific name	No. of protein clusters
Escherichia virus T4	10665	Escherichia virus T4	39
Human immunodeficiency virus 1	11676	Human immunodeficiency virus 1	23
Severe acute respiratory syndrome-related coronavirus	2697049	Severe acute respiratory syndrome coronavirus 2	16
Vaccinia virus	10245	Vaccinia virus	13
Acanthamoeba polyphaga mimivirus	212035	Acanthamoeba polyphaga mimivirus	12
Influenza A virus	11320	Influenza A virus	11

**Table 3:** The list of drugs in complexes with viral proteins in virusMED

Family	Species	Drug name	No. of compounds
*Caliciviridae*	Norwalk virus	Fluorouracil, ribavirin	6
*Coronaviridae*	Severe acute respiratory syndrome-related coronavirus	Chlorzoxazone, clonidine, dalfampridine, histamine, ifenprodil, kinetin, masitinib, nicotinamide, pyrazinamide, salicylamide, sulfapyridine, tipiracil	17
*Filoviridae*	Zaire ebolavirus	Benzatropine, bepridil, clomipramine, ibuprofen, imipramine, paroxetine, sertraline, thioridazine, toremifene	11
*Flaviviridae*	Hepacivirus C	Asunaprevir, grazoprevir, simeprevir	28
*Herpesviridae*	Human α/γ-herpesvirus 8	Acyclovir, ganciclovir, idoxuridine, penciclovir	10
*Myoviridae*	Escherichia virus T4/RB43/RB69	Glucosamine, foscarnet, guaiacol, phylloquinone, toluene	10
*Nimaviridae*	White spot syndrome virus	Methotrexate	2
*Orthomyxoviridae*	Influenza A/B virus	Peramivir, rimantadine, taurine, zanamivir	84
*Paramyxoviridae*	Human respirovirus 3	Zanamivir	2
*Poxviridae*	Vaccinia virus	Cysteamine, rifabutin, rifampicin, rifapentine, rifaximin	5
*Retroviridae*	HIV-1/HIV-2, Western chimpanzee simian foamy virus, Simian/Feline immunodeficiency virus, Murine leukemia virus, Primate T-lymphotropic virus 1	Acepromazine, amphetamine, amprenavir, atazanavir, delavirdine, dolutegravir, doravirine, efavirenz, elvitegravir, etravirine, foscarnet, indinavir, lopinavir, nelfinavir, nevirapine, raltegravir, rilpivirine, ritonavir, saquinavir, tipranavir	211
*Togaviridae*	Aura, Sindbis virus	Piperazine	5
