# Supplementary material for: virusMED: an atlas of hotspots of viral proteins
Source: IUCrJ. 2021 Sep 28;8(Pt 6):931–42. doi: 10.1107/S2052252521009076 (PMC8479994; doi:10.1107/S2052252521009076)
Supplement: Supplementary file 1 [file m-08-00931-sup1.pdf]

IUCrJ

Volume 8 (2021)

Supporting information for article:

VirusMED: an atlas of hotspots of viral proteins

HuiHui Zhang, Pei Chen, Haojie Ma, Magdalena Woinska, Dejian Liu, David Cooper, Guo Peng, Yousong Peng, Lei Deng, Wladek Minor and Heping Zheng

Table S1 The comparison of virusMED with other resources.

	**MetalPDB**	**ZincBind**	**IEDB**	**CED**	**EPIMHC**	**Epitome**	**BindingDB**	**PSMDB**	**VIPERdb**	**virusMED**
**Data source**										
from primary database	✓	✓				✓		✓	✓	✓
use data from UniProt	✓		✓				✓		✓	✓
use data from ICTV									✓	✓
from literature			✓	✓	✓		✓		✓	
external links to primary databases	✓		✓				✓		✓	✓
contains structural data	✓	✓	✓	✓		✓		✓	✓	✓
contains interaction data			✓			✓				✓
**Representation and annotation**										
use 3D structures	✓	✓	✓				✓	✓	✓	✓
hotspot structural annotation	✓	✓	✓				✓	✓		✓
functional sites annotation	✓		✓	✓			✓			✓
prediction of functional sites			✓		✓	✓	✓			
**Types of hotspots**										
metal binding sites	✓	✓								✓
antibody and B cell epitopes			✓			✓				✓
T cell epitopes and MHC			✓		✓					✓
conformational epitope			✓	✓						✓
protein-ligand structures								✓	✓	✓
drugs interactions with their targets							✓			✓
cluster hotspots										✓

Table S2 The list of drugs in complex with viral proteins in virusMED.

family	species	drug_name	component_name
Caliciviridae	Norwalk virus	Fluorouracil, Ribavirin	RNA Dependent RNA Polymerase
Coronaviridae	Severe acute respiratory syndrome-related coronavirus	Chlorzoxazone, Dalfampridine, Histamine, Kinetin, Nicotinamide, Pyrazinamide, Salicylamide	Non-Structural Protein 3
		Clonidine, Ifenprodil, Masitinib, Sulfapyridine	3c-Like Proteinase
		Tipiracil	Uridylate-Specific Endoribonuclease
Filoviridae	Zaire ebolavirus	Benzatropine, Bepridil, Paroxetine, Sertraline	Envelope Glycoprotein,Virion Spike Glycoprotein, Ebov-Gp1
		Clomipramine, Imipramine, Thioridazine	Ebola Surface Glycoprotein, Gp1
		Ibuprofen, Toremifene	Envelope Glycoprotein
		Benzatropine	Virion Spike Glycoprotein, Virion Spike Glycoprotein,Ebola Virus (Makona) Gp2
Flaviviridae	Hepacivirus C	Asunaprevir, Grazoprevir, Simeprevir	Non-Structural Protein 4a, Serine Protease Ns3
		Asunaprevir, Grazoprevir	Ns3 Protease
Herpesviridae	Human α-herpesvirus 8	Acyclovir, Ganciclovir, Idoxuridine, Penciclovir	Thymidine Kinase
	Human γ-herpesvirus 8	Raltitrexed	Thymidylate Synthase
Myoviridae	Escherichia virus T4	Glucosamine, Guaiacol, Toluene	Tail-Associated Lysozyme
		Toluene	Lysozyme
	Escherichia virus RB69	Foscarnet	Dna Polymerase
	Escherichia virus RB43	Phylloquinone	Vitamin K Epoxide Reductase-Like Protein, Termini Restrained By Green Fluorescent Protein
Nimaviridae	White spot syndrome virus	Methotrexate	Thymidylate Synthase
Orthomyxo-viridae	Influenza A virus	Phentermine, Rimantadine	Matrix Protein 2
		Peramivir, Zanamivir	Neuraminidase
		Taurine	Hemagglutinin

	Influenza B virus	Peramivir, Zanamivir	Neuraminidase
Paramyxo-viridae	Human respirovirus 3	Zanamivir	Hemagglutinin-Neuraminidase
Poxviridae	Vaccinia virus	Rifabutin, Rifampicin, Rifapentine, Rifaximin	Scaffold Protein D13
		Cysteamine	Envelope Protein H3
Retroviridae	Human immunodeficiency virus 1	Amprenavir, Atazanavir, Indinavir, Lopinavir, Nelfinavir, Oxygen, Ritonavir, Saquinavir, Tipranavir	Protease, Tethered Dimer
		Delavirdine, Doravirine, Efavirenz, Etravirine, Foscarnet, Nevirapine, Rilpivirine	Reverse Transcriptase/Ribonuclease H
		Amphetamine, Amprenavir, Lopinavir, Saquinavir	Protease
		Acepromazine	HIV-1 trans activating region RNA
		Dolutegravir	Integrase
	Human immunodeficiency virus 2	Amprenavir, Indinavir	Protease
	Western chimpanzee simian foamy virus	Dolutegravir, Elvitegravir, Raltegravir	Pfv Integrase
	Murine leukemia virus	Amprenavir	Gag-Pro-Pol Polyprotein
	Primate T-lymphotropic virus 1	Indinavir	Protease
	Simian immunodeficiency virus	Dolutegravir	Pol Protein
	Feline immunodeficiency virus	Lopinavir	Feline Immunodeficiency Virus Protease
Togaviridae	Aura virus	Piperazine	Capsid Protein
	Sindbis virus	Formaldehyde	Capsid
